# Supplementary material for: Sustainable irrigation based on co-regulation of soil water supply and atmospheric evaporative demand
Source: Nat Commun. 2021 Sep 20;12:5549. doi: 10.1038/s41467-021-25254-7 (PMC8452748; doi:10.1038/s41467-021-25254-7)
Supplement: Supplementary file 1 — Supplementary information [file 41467_2021_25254_MOESM1_ESM.pdf]

Sustainable irrigation based on co-regulation of soil water supply and atmospheric evaporative demand

Jingwen Zhang[1,2*], Kaiyu Guan[1,2,3*], Bin Peng[1,2,3*], Ming Pan[4,5], Wang Zhou[1,2], Chongya Jiang[1,2], Hyungsuk Kimm[1,2], Trenton E. Franz[6], Robert F. Grant[7], Yi Yang[1,2], Daran R. Rudnick[8], Derek M. Heeren[8], Andrew E. Suyker[6], William L. Bauerle[9], and Grace L. Miner[10]

[1]Agroecosystem Sustainability Center, Institute for Sustainability, Energy, and Environment, University of Illinois at Urbana Champaign, Urbana, IL, USA
[2]College of Agricultural, Consumer and Environmental Sciences, University of Illinois at Urbana Champaign, Urbana, IL, USA
[3]National Center for Supercomputing Applications, University of Illinois at Urbana Champaign, Urbana, IL, USA
[4]Department of Civil and Environmental Engineering, Princeton University, Princeton, NJ, USA
[5]Center for Western Weather and Water Extremes, Scripps Institution of Oceanography, University of California San Diego, La Jolla, CA, USA
[6]School of Natural Resources, University of Nebraska-Lincoln, Lincoln, NE, USA
[7]Department of Renewable Resources, University of Alberta, Edmonton, Alberta, Canada
[8]Department of Biological Systems Engineering, University of Nebraska-Lincoln, Lincoln, NE, USA
[9]Department of Horticulture and Landscape Architecture, Colorado State University, Fort Collins, CO, USA
[10]Soil Management and Sugarbeet Research Unit, USDA-ARS, Fort Collins, CO, USA

Corresponding author: Jingwen Zhang (jingwenz@illinois.edu), Kaiyu Guan (kaiyug@illinois.edu), Bin Peng (binpeng@illinois.edu)

Supplementary Tables：

Table S1. Site information under current climate conditions (2001-2019).

Site	Aridity index	MAP (May-Oct)	MAT (May-Oct)	Latitude	Longitude	Altitude (m)	Sand fraction (%)
Mead	1.38	601.69	20.97	41.18	-96.44	359.00	10.0
Harvard	1.62	541.11	20.95	40.65	-98.15	550.00	27.0
GD	1.44	554.73	21.25	40.93	-97.46	494.00	12.0
Lowell	1.23	517.79	21.40	40.62	-98.78	635.00	99.0
Sheridan	1.66	486.95	21.07	40.51	-99.40	715.00	15.0
Dawson	1.65	451.66	20.23	41.02	-100.10	815.00	27.0
NPL	1.81	433.10	20.38	41.09	-100.78	863.00	44.0
PH3	1.73	442.56	20.35	41.06	-101.10	951.00	87.0
T1S4	2.18	359.62	20.33	41.07	-101.85	1010.00	22.0
T1S1	2.22	350.15	20.43	41.00	-102.11	1076.00	77.0
EastBayard	2.14	311.91	19.82	41.78	-103.23	1179.00	76.0
Mitchell	2.14	278.80	19.58	42.02	-103.76	1277.00	87.0

Table S2. Universal and site-specific optimized parameters of SDD and MAD irrigation schemes under current climate (2001-2019) and RCP-8.5 scenario (2058-2076).

Climate conditions	Scenarios		SDD		MAD
	Variables		MAD (%)	Critical G_s (m/s)	MAD (%)
Current (2001-2019)	Site-specific	Mead	45	0.0050	60
		GD	35	0.0070	55
		Harvard	55	0.0055	60
		Sheridan	40	0.0055	55
		Dawson	55	0.0070	60
		NPL	30	0.0055	60
		PH3	50	0.0060	55
		T1S4	45	0.0050	60
	Universal function		50	0.0040	60
RCP-8.5 (2058-2076)	Universal function		65	0.0020	60

Table S3. Parameters in the assessment module (metric unit).

Parameter	Description	Source	Value
y	Maize yield (t/ha)	*ecosys* model simulation	-
p_{maize}	Price of maize ($/t)	USDA NASS (https://www.nass.usda.gov/Quick_Stats/Lite/index.php)	149.99
I	Irrigation amount (mm)	*ecosys* model simulation	-
$\Gamma_{irrigation}$	Cost of irrigation costs, including fuel and labor ($/m^3)	2019 Nebraska Crop Budgets (https://cropwatch.unl.edu/budgets)	0.095
λ	Irrigation application efficiency of the center pivots	U. S. Government Accountability Office (https://www.gao.gov/products/GAO-20-128SP)	0.85
K_{fixed}	Fixed costs of production, including seed, fertilizer, herbicide, crop insurance, and so on ($/ha)	2019 Nebraska Crop Budgets (https://cropwatch.unl.edu/budgets)	1247.86

Table S4. List of 15 Coupled Model Intercomparison Project phase 5 (CMIP5) models under Representative Concentration Pathway 8.5 (RCP-8.5) scenario.

CMIP5 Model ID	Country of origin	Institute
CanESM2	Canada	CCCMA
ACCESS1.0	Australia	CSIRO-BOM
IPSL-CM5A-MR	France	IPSL
MIROC5	Japan	JAMSTEC
MPI-ESM-LR	Germany	MPI-N
CCSM4	USA	NCAR
HadGEM2-ES	UK	MOHC
CNRM-CM5	France	CNRM-CERFACS
CSIRO Mk 3.6	Australia	CSIRO-QCCCE
GFDL-CM3	USA	NOAA, GFDL
INM-CM4	Russia	INM
MRI-CGCM3	Japan	MRI
MIROC-ESM	Japan	JAMSTEC
CESM1-CAM5	USA	NSF-DOE-NCAR
GISS-E2R	USA	NASA/GISS, NY

Table S5. The statistics indexes of *ecosys* simulated daily and monthly GPP, ET, LAI, and yield with flux towers/CPS observations with the maize cropping systems at three AmeriFlux sites (US-Ne1, US-Ne2, US-Ne3) during the growing seasons (May to October) of 2001-2012.

Variables	Indexes		Daily	Monthly
GPP	RMSE (gC/m^2)		3.32	77.58
	NRMSE (%)		34.40	29.30
	Bias (gC/m^2)		0.17	4.58
	NBias (%)		1.80	1.70
	R^2		0.87	0.91
	Slope		0.97	1.01
	Intercept		0.51	0.98
	H0: Slope = 1	F value	$36.47^{*} < F_{(0.05,4300,1)} = 254.31$	$0.26^{*} < F_{(0.05,152,1)} = 254.31$
		p-value	<0.05	0.61^{*}
	H0: Intercept = 0	F value	$46.18^{*} < F_{(0.05,4300,1)} = 254.31$	$0.01^{*} < F_{(0.05,152,1)} = 254.31$
		p-value	<0.05	0.92^{*}
	H0: Slope = 1 and intercept =0	F value	$24.19 > F_{(0.05,4300,2)} = 19.50$	$0.40^{*} < F_{(0.05,152,2)} = 19.50$
		p-value	<0.05	0.67^{*}
ET	RMSE (gC/m^2)		0.93	19.32
	NRMSE (%)		31.30	21.70
	Bias (gC/m^2)		-0.35	-11.21
	NBias (%)		-11.90	-12.60
	R^2		0.80	0.88
	Slope		0.87	0.88
	Intercept		0.03	-0.34
	H0: Slope = 1	F value	$413.04 > F_{(0.05,4690,1)} = 254.31$	$21.88^{*} < F_{(0.05,154,1)} = 254.31$
		p-value	<0.05	<0.05
	H0: Intercept = 0	F value	$2.13^{*} < F_{(0.05,4690,1)} = 254.31$	$0.02^{*} < F_{(0.05,154,1)} = 254.31$
		p-value	0.15^{*}	0.90^{*}
	H0: Slope = 1 and intercept =0	F value	$639.93 > F_{(0.05,4690,2)} = 19.50$	$55.57 > F_{(0.05,154,2)} = 19.50$
		p-value	<0.05	<0.05
LAI	RMSE (gC/m^2)		1.01	0.91
	NRMSE (%)		35.80	35.90
	Bias (gC/m^2)		-0.45	-0.46
	NBias (%)		-15.90	-18.00
	R^2		0.82	0.85
	Slope		0.78	0.78
	Intercept		0.18	0.09
	H0: Slope = 1	F value	$99.02^{*} < F_{(0.05,267,1)} = 254.31$	$44.62^{*} < F_{(0.05,107,1)} = 253.02$
		p-value	<0.05	<0.05
	H0: Intercept = 0	F value	$5.14^{*} < F_{(0.05,267,1)} = 254.31$	$0.75^{*} < F_{(0.05,107,1)} = 253.02$
		p-value	0.03	0.39^{*}
	H0: Slope = 1 and intercept =0	F value	$94.51 > F_{(0.05,267,2)} = 19.50$	$47.87 > F_{(0.05,107,2)} = 19.49$
		p-value	<0.05	<0.05
Yield	RMSE (gC/m^2)		1.25	
	NRMSE (%)		12.58	
	Bias (gC/m^2)		0.17	
	NBias (%)		1.67	
	R^2		0.56	
	Slope		1.01	
	Intercept		0.03	
	H0: Slope = 1	F value	$0.01^{*} < F_{(0.05,22,1)} = 248.53$	
		p-value	0.94^{*}	
	H0: Intercept = 0	F value	$0^{*} < F_{(0.05,22,1)} = 248.53$	
		p-value	0.99^{*}	

	H0: Slope = 1 and intercept =0	F value	$0.20^{*} < F_{(0.05,22,2)}$ =19.45
		p-value	0.82*

o_i and s_i are the observations and model simulations, respectively; RMSE is the Root Mean Square Error ($RMSE = \sqrt{\frac{1}{N}\sum_{i=1}^{N}\left(s_i - o_i\right)^2}$); NRMSE is the Normalized Root Mean Square Error ($NRMSE = \frac{\sqrt{\frac{1}{N}\sum_{i=1}^{N}\left(s_i - o_i\right)^2}}{\overline{o}_i}$); Bias is the mean bias ($Bias = \frac{1}{N}\sum_{i=1}^{N}\left(s_i - o_i\right)$); NBiase is the normalized mean bias ($NBias = \frac{\frac{1}{N}\sum_{i=1}^{N}\left(s_i - o_i\right)}{\overline{o}_i}$); R^2 is the coefficient of determination ($R^2 = 1 - \frac{\sum_{i=1}^{N}\left(s_i - o_i\right)^2}{\sum_{i=1}^{N}\left(s_i - \overline{o}_i\right)^2}$). H0: Slope = 1 denotes the null hypothesis of slope equals 1 with no constraints on the intercept. H0: Intercept = 0 denotes the null hypothesis of intercept equals 0 with no constraints on the slope. H0: Slope = 1 and intercept =0 denotes the null hypothesis of slope and intercept equal 1 and 0, respectively. The F-test is applied to investigate whether the regression coefficients equal the given parameters significantly or not (slope equals 1 and intercept equals 0). If the p-value from the F-test is less than the given significance level ($\alpha = 0.05$) and the F-value from the F-test is larger than the critical value in the F distribution with given significance level ($\alpha = 0.05$), the null hypothesis can be rejected, denoting that the regression coefficients equal the given parameters insignificantly. If we fail to reject the null hypothesis, we accept it by default, denoting that the regression coefficients equal the given parameters significantly (denoted with * in Table S5). $F_{(\alpha, df_1, df_2)}$ denotes the critical value in the F distribution with a given significance level ($\alpha = 0.05$) with denominator degrees of freedom (df_1) and numerator degrees of freedom (df_2), which could be obtained from F distribution tables.

Supplementary Figures:

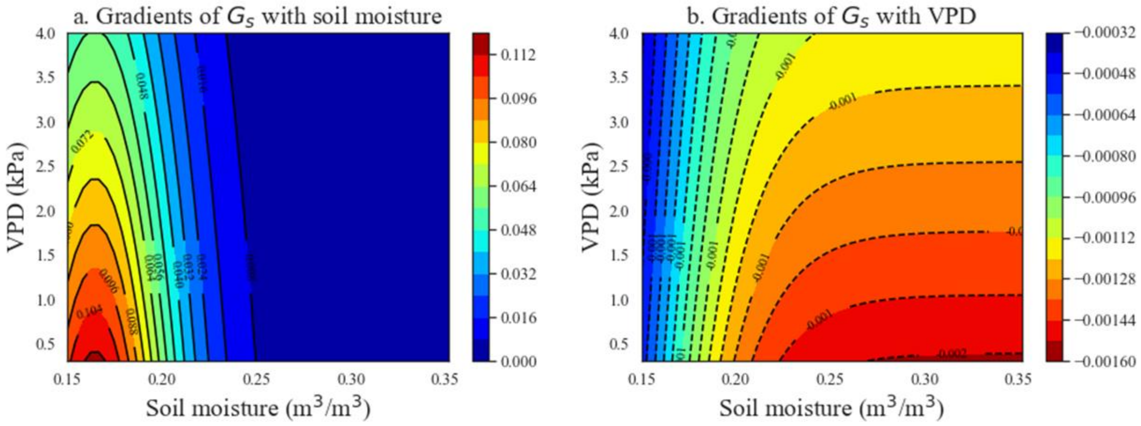

Fig. S1 Gradients of simulated canopy-level stomatal conductance (G_s) with **a**, soil moisture and **b**, VPD at the daily scale based on the *ecosys* model under continuous maize cropping systems during peak growing seasons (July and August) under current climate (2001-2019) at site-GD (40.93°N, 97.46°W) in Nebraska.

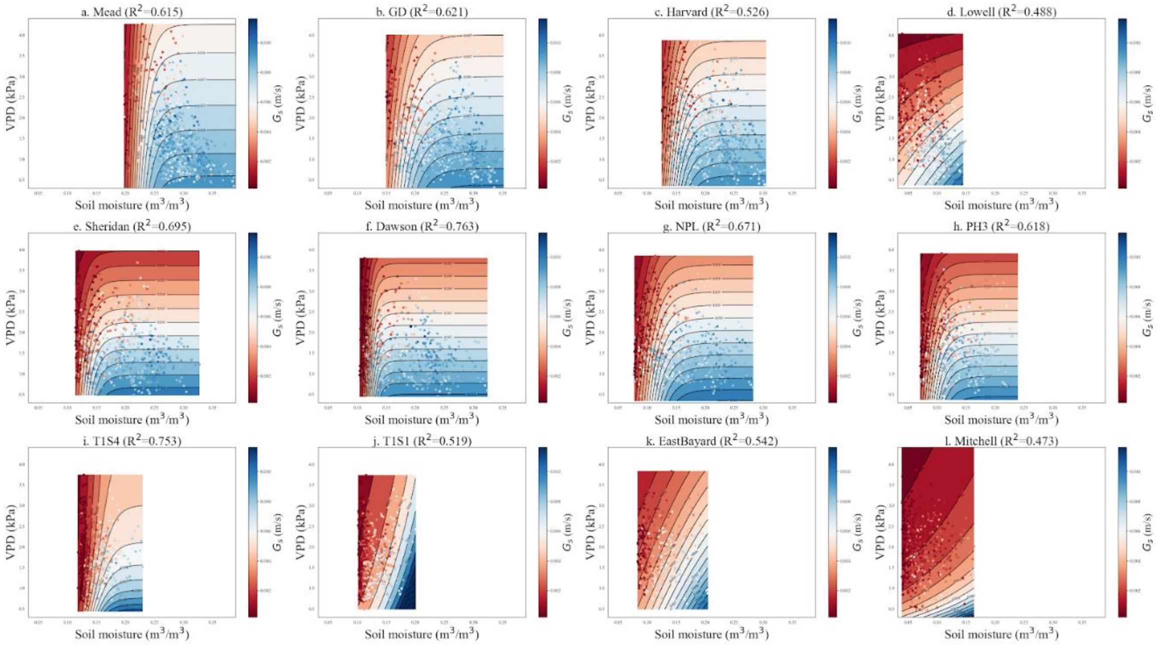

Fig. S2 Fitted contours of G_s under the co-regulation of soil moisture (the top 9 soil layers with a depth of 0.92 m) and VPD based on simulations from *ecosys* model during the peak growing season across 12 sites in Nebraska under current climate (2001-2019). The high performance of the regression further confirmed the co-regulation of soil moisture and VPD on G_s, except sites-Lowell, T1S1, Eastbayard, and Mitchell under extreme dry climate or sandy soil conditions (**d**, **j**, **k**, and **l**).

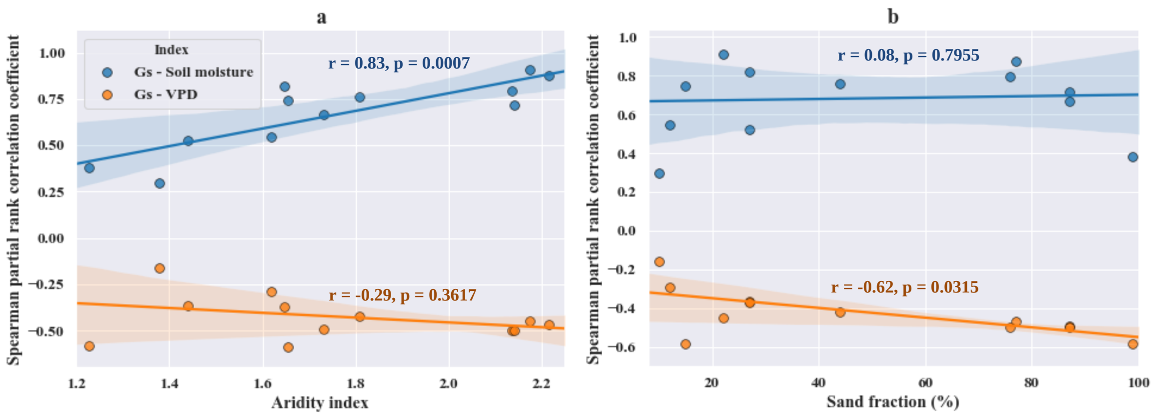

Fig. S3 Variation of the Spearman partial rank correlation coefficient (p-value of each point was less than 0.001) between G_s and soil moisture/VPD with **a**, aridity index and **b**, sand fraction across 12 sites in Nebraska under current climate (2001-2019). The line denoted the regression line with a 95% confidence interval. r and p denoted the correlation coefficient and p-value, respectively.

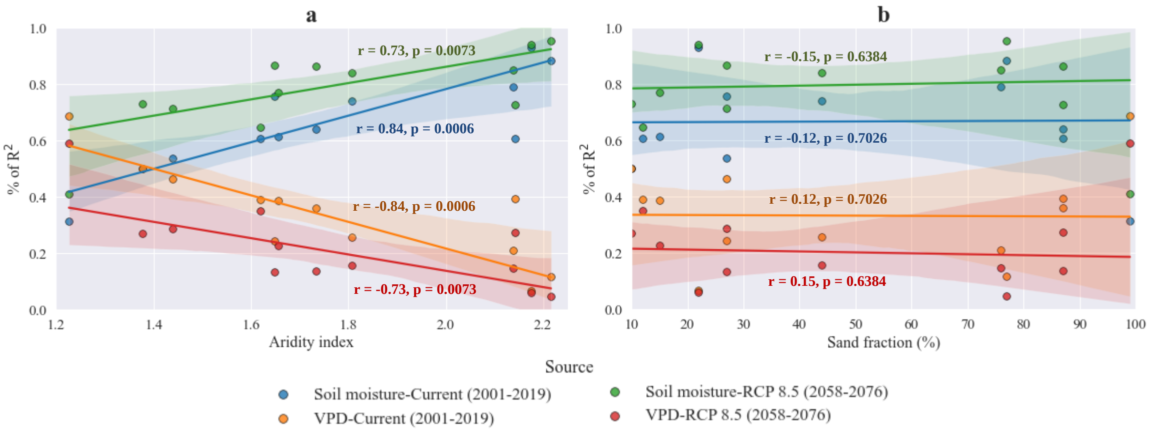

Fig. S4 Variation of the relative importance of soil moisture and VPD on G_s with **a**, aridity index and **b**, sand fraction across 12 sites in Nebraska under current climate (2001-2019) and RCP-8.5 scenario (2058-2076). The line denoted the regression line with a 95% confidence interval. r and p denoted the correlation coefficient and p-value, respectively.

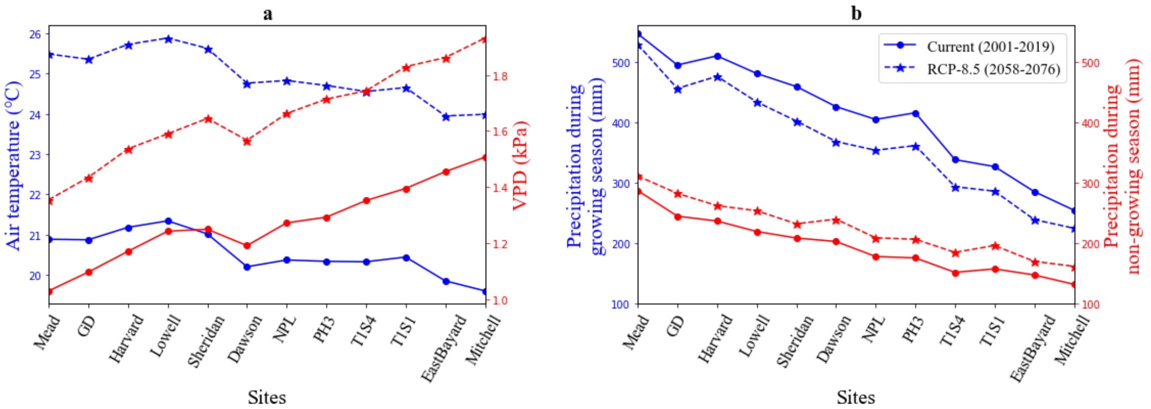

Fig. S5. Current and projected **a**, air temperature and VPD during growing season (defined in Methods), and **b**, precipitation during growing and non-growing season across 12 sites in Nebraska under current climate (2001-2019) (solid curves with dots) and RCP-8.5 scenario (2058-2076) (dashed curves with stars).

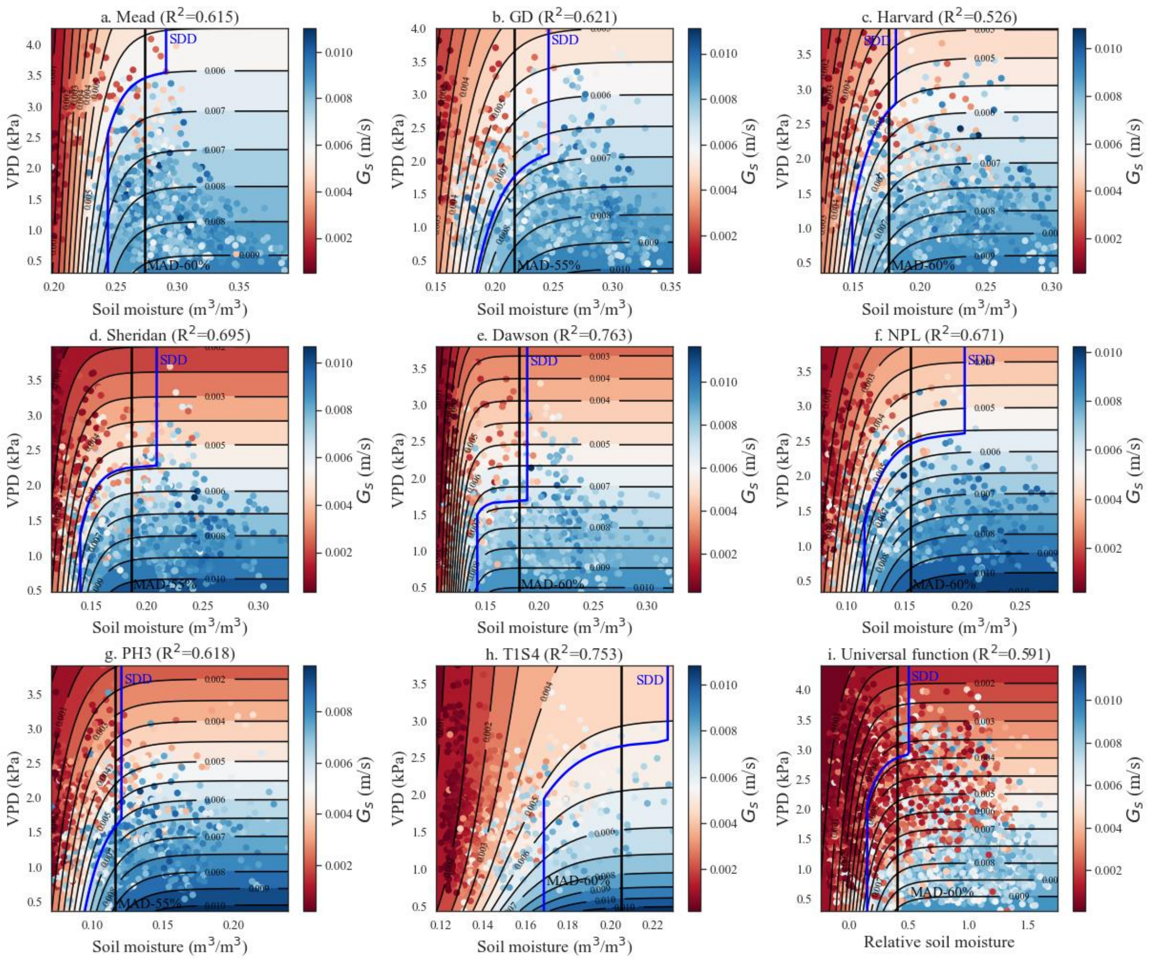

Fig. S6 Comparison of SDD and MAD irrigation schemes with site-specific and universal parameters under current climate (2001-2019). The blue and black curves were the site-specific thresholds at 8 sites (**a** to **h**) and universal thresholds (**i**) based on the scatter and contour of G_s based on the co-regulation from soil moisture and VPD for maize cropping systems in Nebraska.

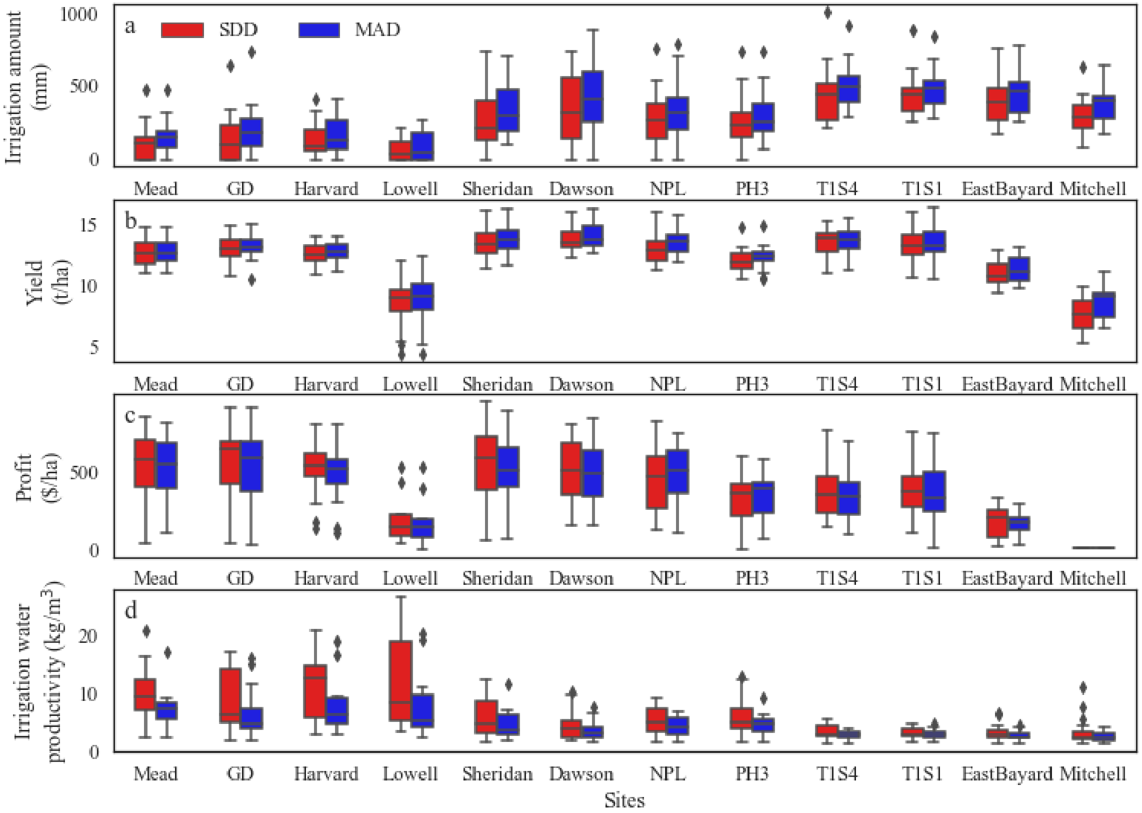

Fig. S7 Box plots of **a**, irrigation amount, **b**, yield, **c**, profit, and **d**, irrigation water productivity of SDD and MAD irrigation schemes with universal parameters under current climate (2001-2019) across 12 sites in Nebraska. Each box represented the 25th and 75th percentiles, and the outliers were marked using black diamonds.

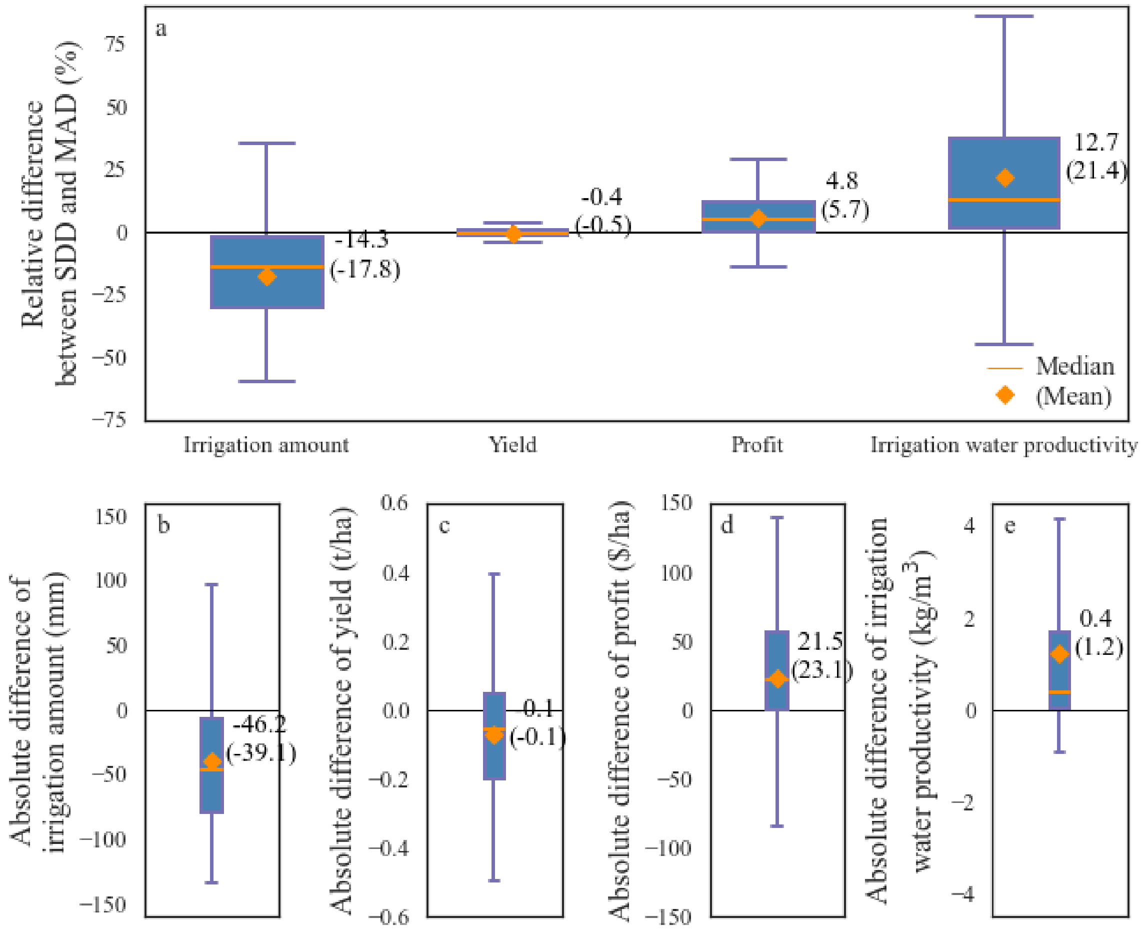

Fig. S8 Performances of plant-centric SDD and soil-moisture-based MAD irrigation schemes under current climate (2001-2019). Relative (**a**) and absolute (**b**, **c**, **d**, and **e**) differences in irrigation amount, yield, profit, and irrigation water productivity between SDD and MAD irrigation schemes with site-specific parameters across 8 sites (sites-Mead, GD, Harvard, Sheridan, Dawson, NPL, PH3, and T1S4) in Nebraska under current climate (2001-2019). Boxes showed 25th–75th percentiles, and the orange line and diamond denoted the median and mean for each box, respectively.

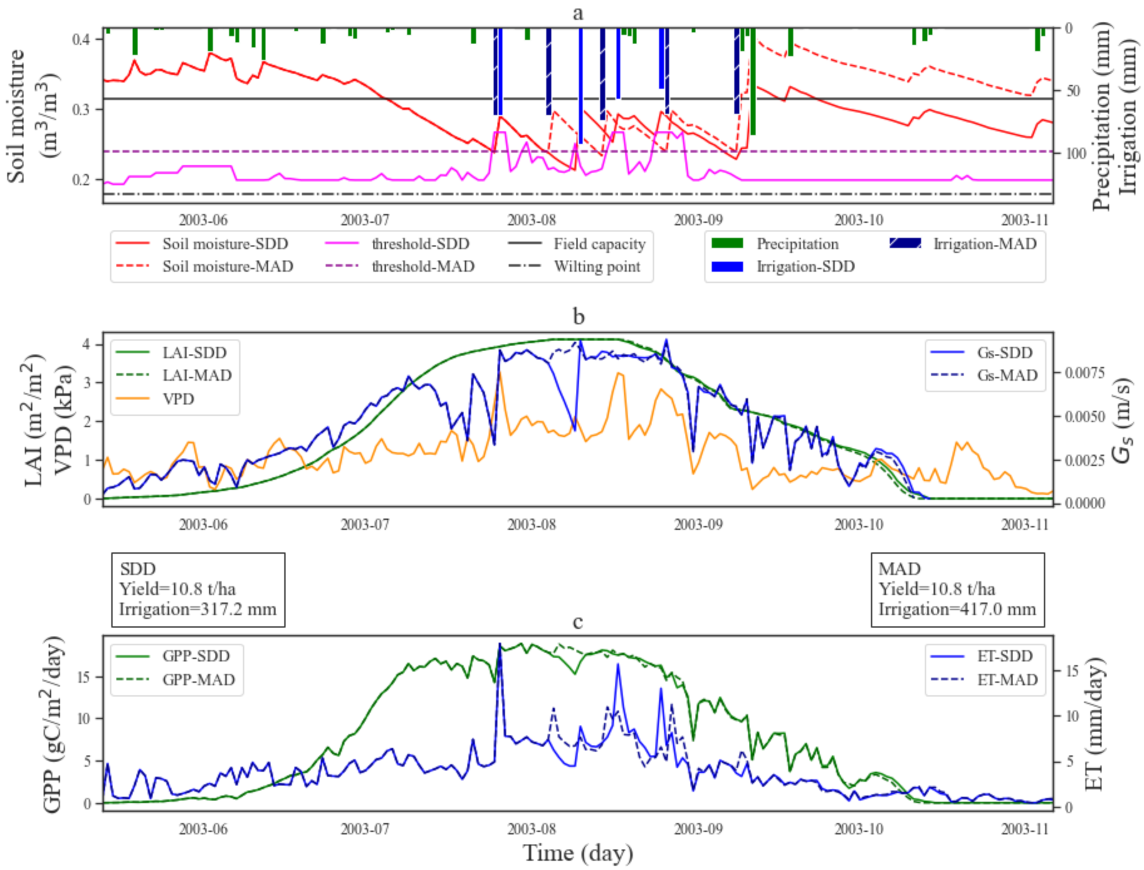

Fig. S9 Comparison of the performances between SDD and MAD irrigation schemes with site-specific parameters during the growing season at site-GD in 2003. Variation of **a**, soil moisture, soil moisture threshold, precipitation, irrigation; **b**, LAI, VPD, and G_s; and **c**, GPP and ET.

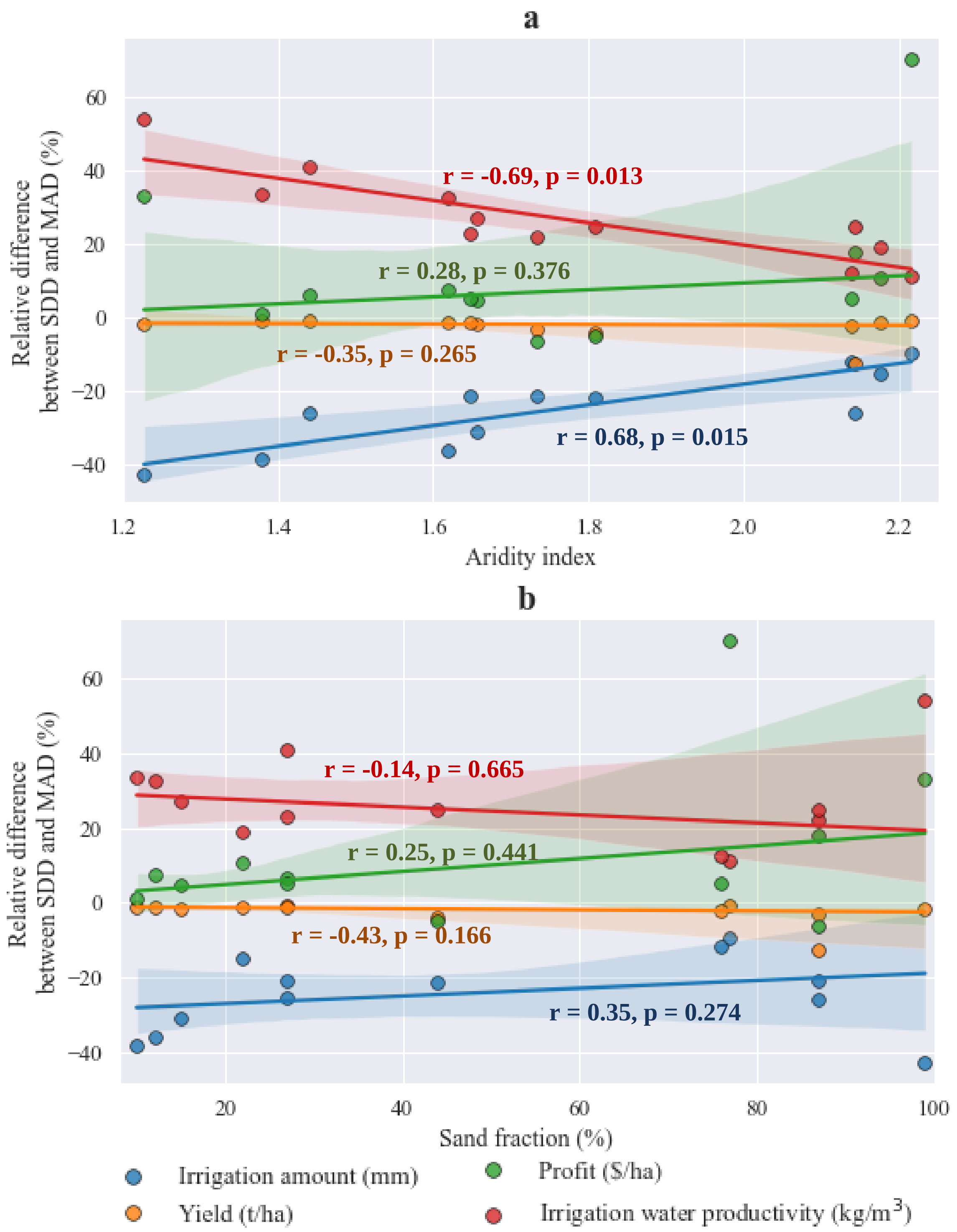

Fig. S10 Variation of relative differences in irrigation amount, yield, profit, and irrigation water productivity between the SDD and MAD irrigation schemes with **a**, aridity index and **b**, sand fraction across 12 sites in Nebraska under current climate (2001-2019).

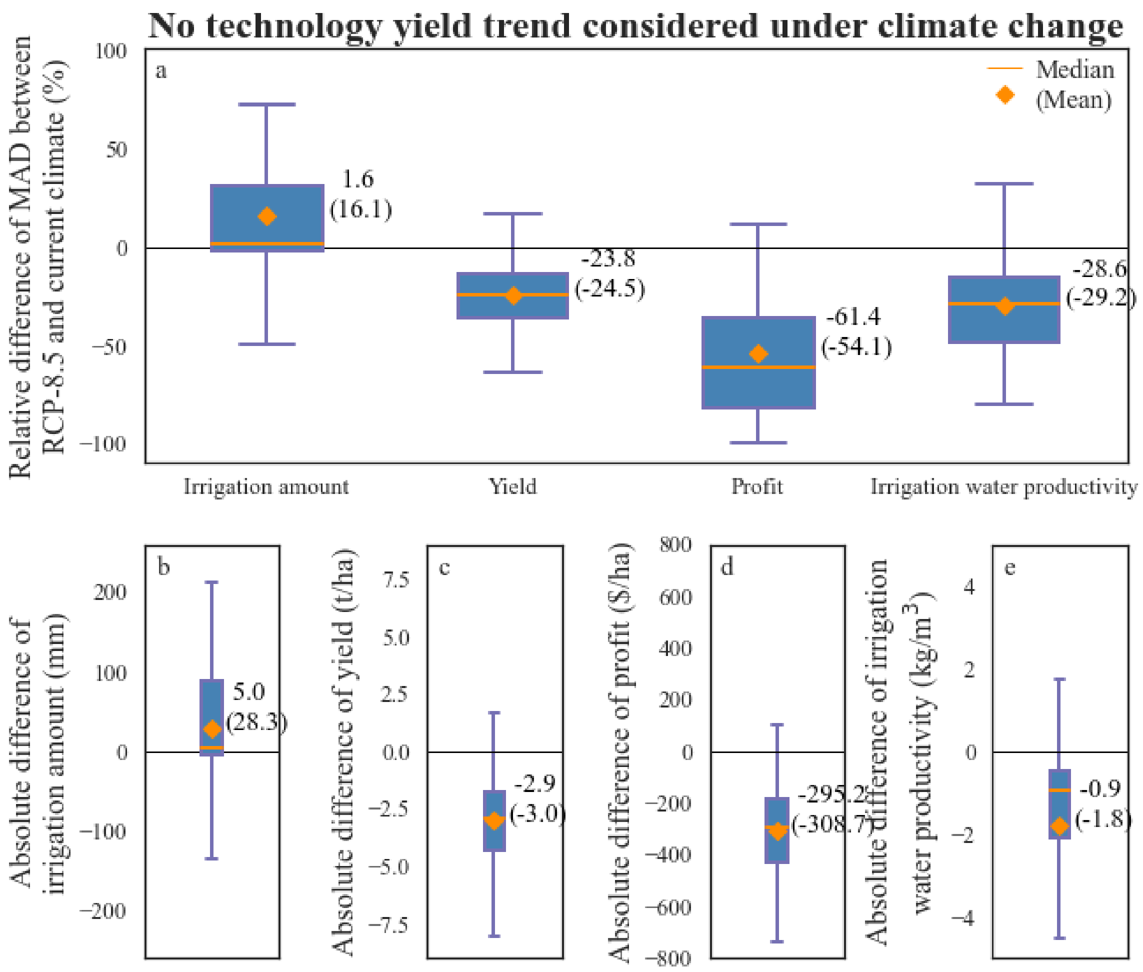

Fig. S11 Performances of MAD irrigation scheme between current climate (2001-2019, baseline) and RCP-8.5 scenario (2058-2076). Relative (**a**) and absolute (**b**, **c**, **d**, and **e**) differences in irrigation amount, yield, profit, and irrigation water productivity with universal parameters between current climate (2001-2019) and RCP-8.5 scenario (2058-2076) across 12 sites in Nebraska.

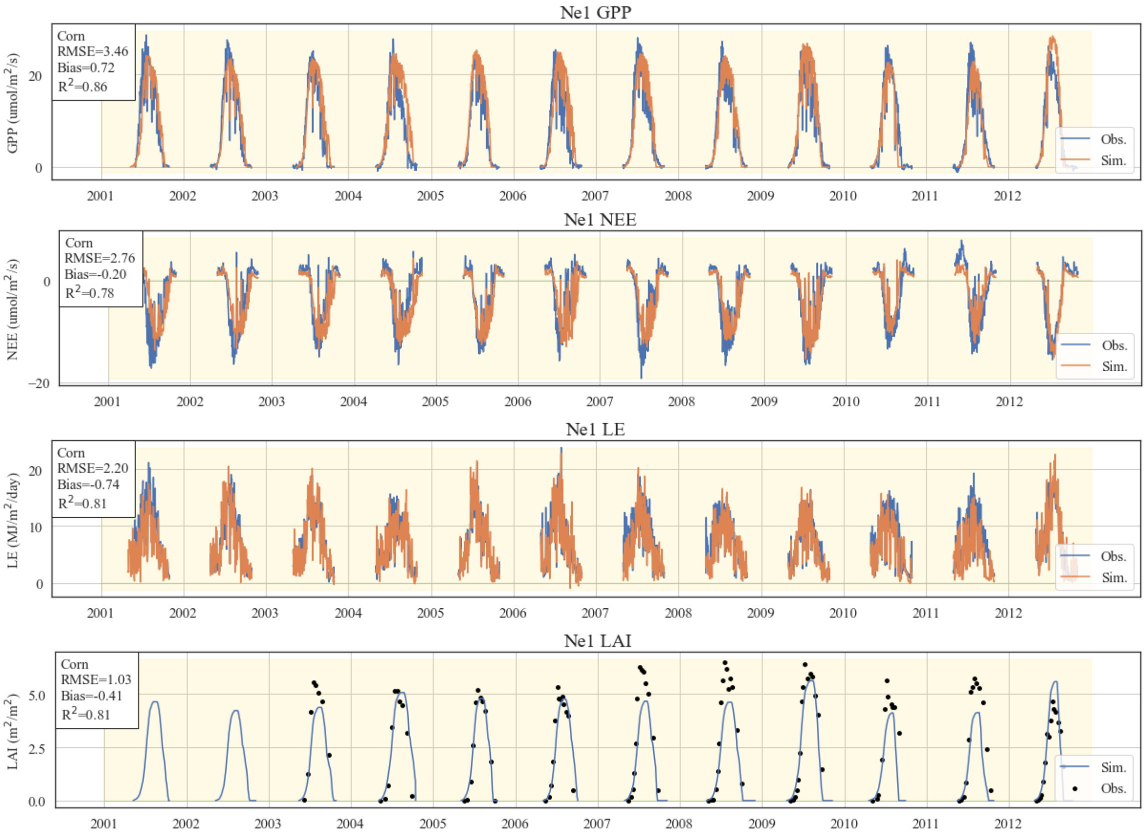

Fig. S12 Performance of *ecosys* model on daily GPP, NEE, LE, and LAI with the statistical results (RMSE, Bias, and R^2) for continuous maize cropping systems during the growing season (May to October) in the periods 2001-2012 at US-Ne1.

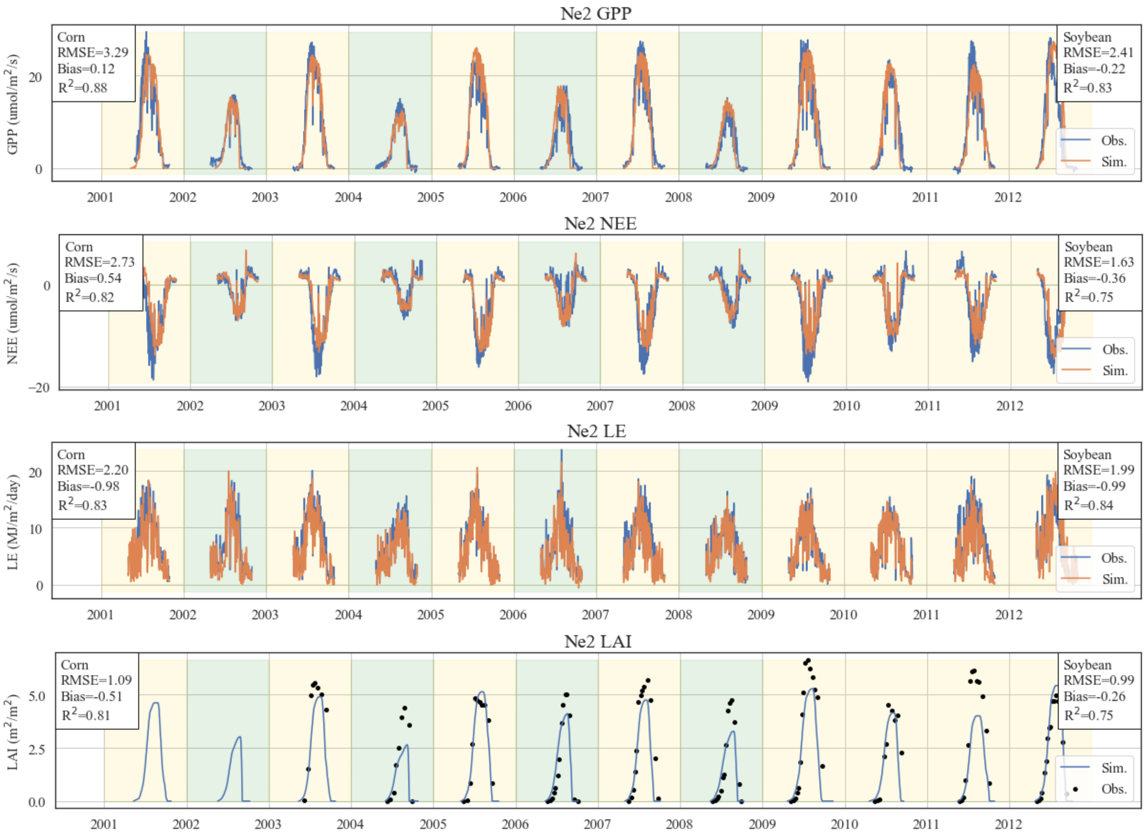

Fig. S13 Performance of *ecosys* model on daily GPP, NEE, LE, and LAI with the statistical results (RMSE, Bias, and R^2) for maize (yellow regions) and soybean (green regions) cropping systems during the growing seasons (May to October) in the period 2001-2012 at US-Ne2.

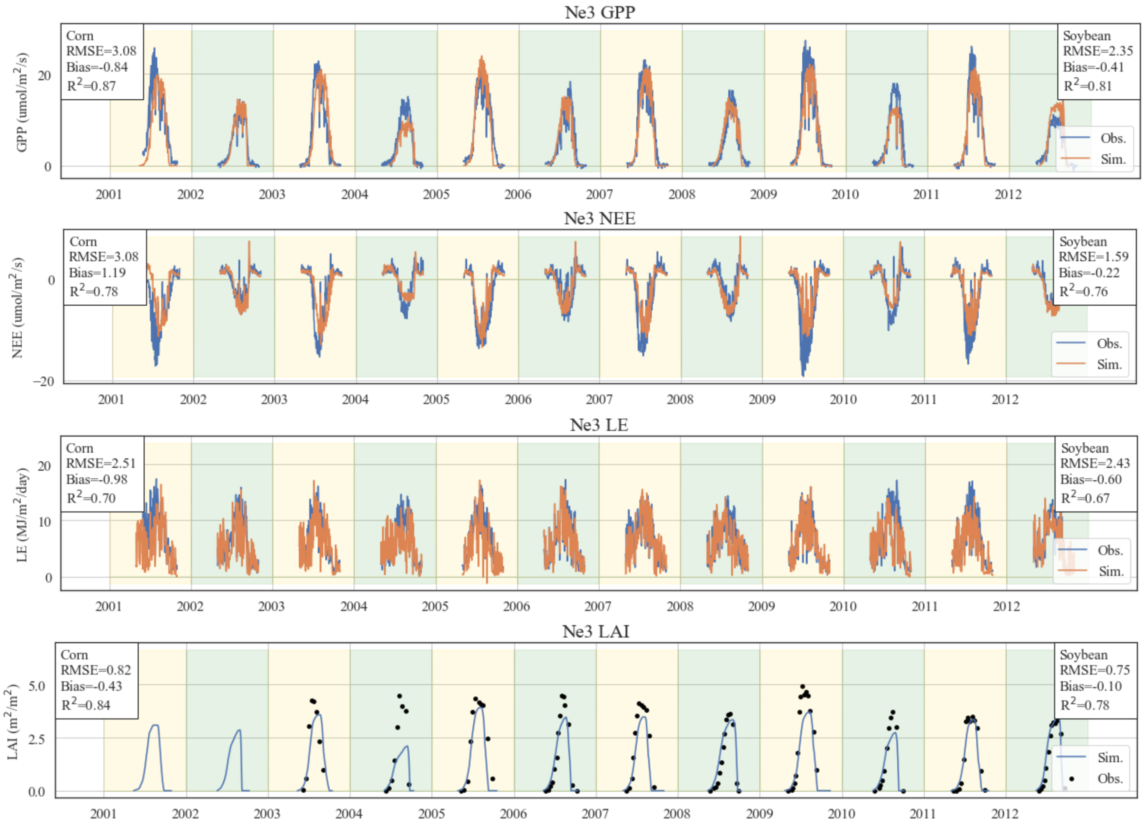

Fig. S14 Performance of *ecosys* model on daily GPP, NEE, LE, and LAI with the statistical results (RMSE, Bias, and R^2) for maize (yellow regions) and soybean (green regions) rotation cropping systems during the growing seasons (May to October) in the period 2001-2012 at US-Ne3.

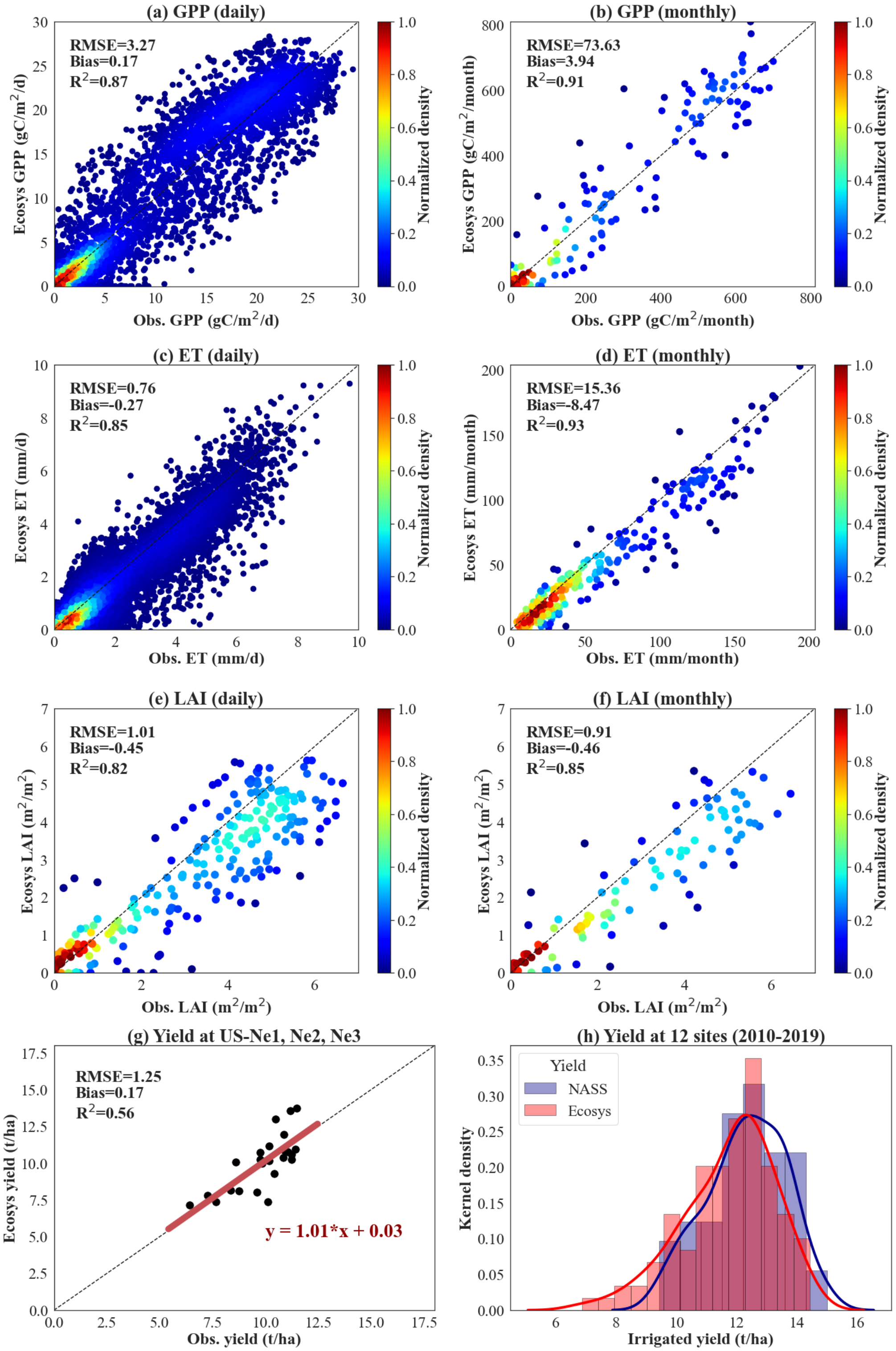

Fig. S15 The performance of *ecosys* model with maize cropping systems at three AmeriFlux sites (US-Ne1, Ne2, and Ne3) during the period from 2001-2012 and 12 sites with a dramatic rainfall gradient across Nebraska during the period from 2010-2019. The colorbar showed the normalized gaussian kernel density estimation of the scatters. Black dashed lines indicated the 1-to-1 relationship. The red line was the regression line with the slope and intercept. The probability density function of the maize yields at the 12 sites was the gaussian kernel density estimation.
